# Yogurt and curd cheese as alternative ingredients to improve the gluten-free breadmaking

**DOI:** 10.3389/fnut.2022.934602

**Published:** 2022-11-04

**Authors:** Carla Graça, Anabela Raymundo, Isabel Sousa

**Affiliations:** ^1^LEAF – Linking Landscape, Environment, Agriculture and Food Research Center of Instituto Superior de Agronomia, Universidade de Lisboa, Lisbon, Portugal; ^2^Department of Food and Nutrition, Faculty of Agriculture and Forestry, University of Helsinki, Helsinki, Finland

**Keywords:** dairy proteins, gluten-free dough, rheology properties, linear correlation, bread quality

## Abstract

Gluten-free products are on today’s agenda since they represent the most hastily growing segments in the market, representing an opportunity for food companies. Nevertheless, it is well-known that gluten is a crucial network structure in the wheat dough systems, which accounts for the overall desired technological features of the final bakery goods. Therefore, the absence of gluten negatively affects the characteristics of gluten-free bread, triggering a technological challenge in the manufacturing of products with resembled characteristics of wheat-derived counterparts. The search for new protein sources has been studied as an approach to circumvent the technological drawbacks of gluten removal. Dairy proteins are functional molecules that can likely be capable of building up a protein-network structure so that it would improve the technological properties of gluten-free products. In the present work, different levels of dairy product addition (10 and 20%, w/w) were used to supplement the gluten-free bread formulas, and the impact on dough rheology properties was well correlated to the bread technological quality parameters obtained. Linear correlations (*R*^2^ > 0.904) between steady shear (viscosity) and oscillatory (elastic and viscous moduli) values of the dough rheology with bread quality parameters (volume and firmness) were obtained, suggesting that the bread quality improvements are proportional to the levels of dairies added. Likewise, strong linear correlations (*R*^2^ > −0.910) between pasting properties parameters and bread staling rate supported the hypothesis that the dairies tested have a high potential to generate bread with a low staling rate, which is an advantage to extending the shelf-life. In short, results confirmed that the addition of both dairy products, as bakery ingredients, can constitute a technological advantage to improve the overall gluten-free bread quality.

## Introduction

The development of gluten-free foods is on today’s agenda, and it has been leading to an increasingly soaring demand for gluten-free products in the market, not only due to the growing number of individuals diagnosed with some type of gluten intolerance but also of those non-celiac consumers focused on healthy food choices. Altogether, it represents around 38% of avoidance and/or limiting of wheat or/and gluten-containing foods (1).

Gluten is a structure-building protein, composed of glutenin and gliadin, both responsible for elasticity and extensibility of the dough, that plays an important role in breadmaking as they are responsible for the high-quality baked products (2). However, in the context of genetically susceptible individuals, such as those suffering from celiac disease, the ingestion of gluten can promote severe and irreversible healthy consequences (3). Currently, the gluten-free (GF) diet is the only option for people suffering from gluten-related disorders.

The manufacturing of gluten-free products, especially bread, represents a technological challenge due to the importance of gluten proteins on dough structure, essential to form a viscoelastic network capable to retain the gases (CO_2_), produced during the baker’s yeast fermentation stage and to support the expansion of the dough-foaming structure. The absence of gluten results in bread with post-baking quality defects such as lower volume and crumbly texture with a fast staling rate, therefore, with poor overall appearance (4).

In those matters, the research of possible alternatives to improve the technological properties of the existing products, by the combination of gluten-free flours with structuring agents to produce a protein-structure like gluten network, is an important and actual branch of research.

Proteins are naturally good hydrocolloids and one of the main molecule classes available to improve desirable textural attributes, promoting crosslinking and aggregation mechanisms, with a great impact on food structuring. They are extensively used in different gluten-free formulas, such as egg albumin and whey protein, due to their technological functionality, based on water-binding and holding capacity, emulsifier, foaming, and gelling properties (4–7).

Dairy products are known as a source of high functionality and versatility proteins since they are capable of being used in a vast number of food manufacturing applications (8), whether for nutritional or functional benefits (9–13). Dairy proteins have been reported to have interesting functional properties such as emulsifying and stabilizing abilities, gelling, and foaming features, high water-absorption, and binding capacity, which make them able of forming a protein network with desirable swelling properties, resembling the gluten network (8). Thanks to the high water-holding capacity of dairy proteins, they might be able to generate a proper batter viscosity as well as boost the protein interaction with more flexible effects. Those properties are crucial to achieving high-quality gluten-free products, especially in breadmaking (2).

Recent research has shown that the incorporation of dairy products (e.g., yogurt) into bakery good formulations was able to improve both the desired bread quality attributes and the nutritional value of gluten-containing (14, 15) and gluten-free bread (16), as well as in sourdough wheat bread (17). Other researchers showed relevant enhancements in breadmaking, by the addition of whey protein to gluten-free bread formulations, reporting the capacity to increase water absorption and improve the handling properties (4). Good thermal gelation capacity of dairy proteins, such as whey protein-inducing viscoelastic gels by protein network, capable to support dough foaming was recently reported (18, 19). Additionally, it was demonstrated that the utilization of whey cheese (a type of cheese made by the heat precipitation of soluble whey proteins) might be an alternative bakery ingredient to face the technological challenges of gluten absence (20) while improving the protein and minerals profile of the gluten-free goods (21).

Yogurt (Yg) is one of the most popular fermented dairy products, characterized by a precipitated casein network, obtained by the acidification activity of *Streptococcus thermophilus* and *Lactobacillus bulgaricus subsp. delbrueckii* cultures (22). Widely consumed and appreciated in the daily diet, with an important role in nutrition, it is a rich source of high-biological protein nutritionally good and easily digestible (23, 24). Additionally, it is a source of exopolysaccharides *in situ* produced by its dominant lactic acid bacteria fermentative activity, that has been reported as an aid-technological baking improver component with healthy benefits (25, 26). Curd cheese (Cc) is a co-product obtained by the thermal denaturation and subsequent precipitation of the soluble whey proteins, a liquid by-product of cheese manufacturing, that is widely accepted for containing many valuable interesting components to improve human nutrition (27).

In these matters, both yogurt and curd cheese can probably be seen as promising bakery ingredients for the development of gluten-free bread technology and nutritional improvement.

This research work was designed to study the technological functionality of the yogurt and curd cheese additions to mimic the gluten-like structure so that it would improve the gluten-free breadmaking performance. Different levels of both dairies (20 g and 40 g/100 g of flour basis, corresponding to 10–20% weight/weight (w/w), in overall percentage), into a gluten-free dough, were tested, and the effect on dough rheology properties, by steady shear flow behavior, and small amplitude oscillatory assays were assessed. The impact on gluten-free bread quality, by evaluating the loaf firmness and staling kinetic during the 4 days of storage time, as well as by the after-baking quality attributes, was also evaluated. Linear correlations were studied among dough rheology and bread quality attributes to acquire additional information about the processes and variables involved in the process.

## Materials and methods

### Raw materials

Gluten-free bread samples were prepared with a mixture of rice (g/100 g: moisture 10.9 g, protein 7.0 g, lipid 1.3 g, carbohydrates 80.0 g, fiber 0.5 g), buckwheat flour (g/100 g: moisture 13.1 g, protein 13.3 g, lipid 3.4 g, carbohydrates 61.5 g, fiber 10.0 g), and potato starch (g/100 g: moisture 17.5 g, protein 0.2 g, lipid 0.1 g, carbohydrates 80.0 g).

The plain yogurt used was a commercial product from LongaVida, Portugal (g/100 g: moisture 88.5 g, protein 3.7 g, lipid 3.7 g, carbohydrates 5.5 g, fiber 0.8 g).

The curd cheese lactose-free used was a commercial product from Lacticínios do Paiva (Lamego, Paiva, Portugal), obtained by thermal precipitation of the whey, resulting from the lactose-free cheese manufacturing (g/100 g: moisture 74.2 g, protein 10.7 g, lipids 9.0 g, fiber 0.2 g, carbohydrates 3.0 g).

Commercial white crystalline saccharose (Sidul, Santa Iria de Azóia, Portugal), sea salt (Vatel, Alverca, Portugal), baker’s dry yeast (Fermipan, Lallemand Iberia, SA, Setúbal, Portugal), vegetable oil (Vegê, Sovena Group, Algês, Portugal), and xanthan gum (Naturefoods, Lisboa, Portugal) were used.

### Bread dough’s preparation and breadmaking

Gluten-free bread dough was prepared as previously described by Graça et al. (20) with some modifications: first, the dry baker’s yeast was activated in warm water in the processor cup, for 2 min at position 3. Afterward, the remaining ingredients were added and homogenized for 1 min at position 6, and the kneading was carried out for 10 min. Xanthan gum was used as a hydrocolloid. A proofing time of 20 min was selected since longer fermentation collapsed the dough structure. Gluten-free bread samples were baked in a convection oven at 180°C for 30 min. The bread formulas tested are summarized in Table 1. Ingredients kept constant: salt, 1.5%; sugar, 2.8%; dry yeast, 2.8%; xanthan gum, 0.5%; and vegetable oil, 5.5% (gluten-free mixture flour basis).

**TABLE 1:** Gluten-free bread formulations of control bread (CB) and breads obtained with different levels of yogurt (YgB) and curd cheese (CcB) additions, considering the dry extract coming from Yg (11.5%) and Cc (31.2%) to replace on flour basis.

Ingredients	CB	YgB10%	YgB20%	CcB10%	CcB20%
Buckwheat	16.6	14.0	11.4	13.6	11.0
Rice	24.8	21.1	17.1	20.4	16.5
Potato starch	13.8	11.7	9.5	11.3	9.2
Yogurt/curd cheese	0.0	10.0	26.7	10.0	16.8
Added water*	37.5	29.0	20.0	32.3	29.4
Yogurt/curd cheese water**	0.0	10.0	14.1	6.6	11.6
Total water absorption***	37.5	39.0	34.1	38.9	41.0

*Determined by mixing curves performed in the MicroDoughLab equipment; **Water coming from Yg and Cc addition; *** Total water absorption = water added + Yg and Cc derived water.

The water absorption of bread samples by applying the MicrodoughLab mixing curves procedure (Perten Instruments, Hägersten, Sweden) was determined as following: fixing the viscosity torque values of the gluten-free control dough (16.0 ± 2.0 milliNewton meter—mNm), previously optimized, the water absorption of the flour mixture was determined at each level of yogurt and curd cheese tested (based on 14% of moisture basis). The yogurt and curd cheese-derived water were considered, at each level tested, to optimize the bread formulas. Replacements were based on gluten-free flour basis as earlier described (20).

### Pasting properties

The effect of yogurt and curd cheese additions on the starch physical behavior of the gluten-free bread dough, in comparison to control dough (CD), was studied by MicrodoughLab measurements (Perten, instruments, Hägersten, Sweden) (15). The method was performed with some modification as follows: heating-cooling cycles were applied, according to the following set of conditions: sample homogenization for 30 s, mixing curve at 30°C for 360 s, heating from 30 to 95°C for 390 s, standing at 95°C for 60 s, and cooling down to 50°C for 390 s, at similar temperature rate (0.17°C/s). The paddle speed was 63 rpm for running the analysis. MicrodoughLab parameters that characterize the consistency of the dough during mixing, heating (cooking), and cooling phases were recorded in torque units (mNm) (AACC, 54–60.01): dough development or maximum torque (C1) was reached during mixing at 30°C, the minimum torque of dough when subjected to mechanical and thermal conditions by heat denaturation of proteins (C2), peak torque of starch gelatinization (C3), cooking stability or minimum torque during the heating period (C4), and final consistency peak torque produced after cooling stage at 50°C (C5). Triplicates were performed.

### Dough rheology measurements

The influence of yogurt and curd cheese additions on dough rheology properties was assessed by following the changes in flow viscosity and viscoelastic profiles, after 20 min of fermentation time. Rheology property assessments were conducted at 5°C of temperature (to inactivate the yeast activity) and carried out in a controlled stress rheometer (Haake Mars III—Thermo Scientific, Karlsruhe, Germany), with a Universal Temperature Control—Peltier system to control temperature, using a serrated parallel plate sensor system (PP35 and 1 mm gap), to overcome the slip effect (28).

The impact on dough flow behavior was registered by steady shear conditions varying the shear rate from 1.0 × 10^–6^ to 1.0 × 10^3^ s^–1^. The Carreau model was used to model the flow curves obtained, applying the Eq. 1:


(1) η=η/0[1+(γ./γc.)]2s

where η is the apparent viscosity (Pa. s), γ. is the shear rate (s^–1^), η_0_ is the zero-shear rate viscosity (Pa. s), γ ._*c*_ is a critical shear rate for the onset of the shear-thinning behavior (s^–1^), that is, the value corresponding to the transition from Newtonian to shear-thinning behavior, and s is a dimensionless parameter related to the slope of this region.

The effect on viscoelastic functions focused on storage (G′) and dissipative (G″) moduli were followed by frequency sweep measurements, ranging the frequency from 0.001 to 100.0 Hz, at constant shear stress (10 Pa), within the linear viscoelastic region of each sample, previously determined (at 1 Hz). Triplicates were performed.

### Gluten-free bread quality assessment

#### Post-baking quality parameters

Derived control and dairy gluten-free bread samples were evaluated based on post-baking quality parameters, such as bread crumb texture and staling aging over the 4 days of storage time, moisture (%), bake loss (BL), and specific bread volume (SBV) (cm^3^/g). Bread crumb firmness progressing over the 4-day storage time was evaluated using a texturometer TA-XTplus (Stable MicroSystems, Surrey, UK) in penetration mode, according to the description of the previous method (14, 20).

The influence of dairy products addition was also evaluated focused on bread staling rate, described as a function of time,


(2) F⁢i⁢r⁢m⁢n⁢e⁢s⁢s=A×t⁢i⁢m⁢e+B

where A can be considered the staling rate and B the initial firmness of the bread.

Bread crumb moisture was determined according to the standard method AACC 44–15.02. Bread volume was measured by the rapeseed displacement standard method AACC 10-05.01, after 2 h of bread cooling down. Specific volume (cm^3^/g) was calculated as the ratio between the bread volume and its weight. The baking loss was assessed by weighing the bread forms before and after baking. Triplicates were performed.

### Statistical analysis

The experimental data were statistically analyzed by determining the average values and standard deviation, at the significance level of 95%, for each parameter evaluated. Statistical analysis (RStudio, Version 1.1.423) was performed by applying variance analysis, the one factor (ANOVA), and *post-hoc* comparisons (Tukey test). Experimental rheology data were fitted to the non-linear Carreau model, using the TA Instruments/TRIOS software.

## Results and discussion

### Pasting properties measurements

The influence of yogurt and curd cheese incorporations on gluten-free dough was evaluated following the changes in pasting parameters by Microdoughlab measurements.

**FIGURE 1:**
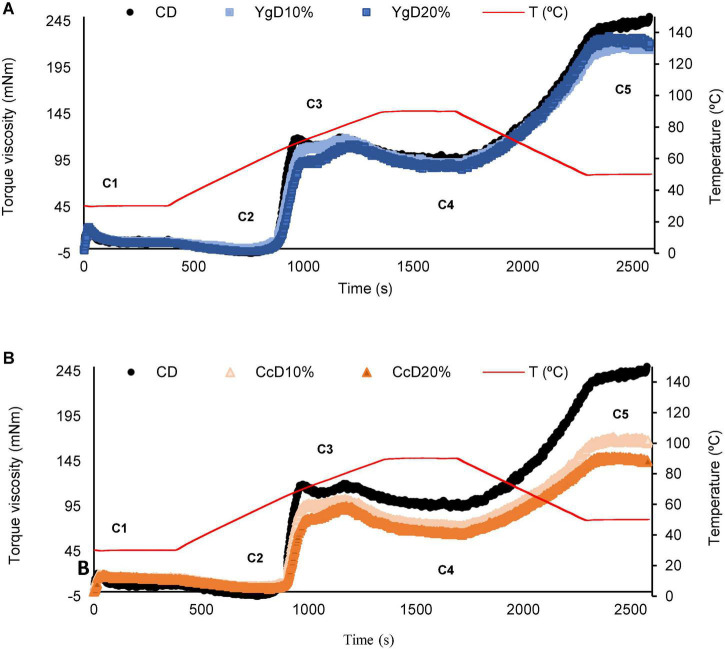
Comparison of pasting properties profile of dairy gluten-free dough, obtained by different levels (10–20%, w/w) of yoghurt **(A)** and curd cheese **(B)** addition, with control bread dough profile (black line).

Figure 1 shows the comparison between the heating-cooling curves obtained for the four-dairy gluten-free dough samples with the control dough sample (no dairies added).

As can be seen, the control and dairy doughs exhibited low consistency at the initial stage (C1) of dough development. These results were expected since the doughs in the study are gluten-free (29). Additionally, as the heating stage increases to about 60°C, the systems tended to phase separation, resulting in a further decrease of dough consistency at the C2 step, referring to protein weakening. This behavior might suggest that the yogurt-derived caseins and exopolysaccharides (EPS) might be acting as lubricants with destabilizing effects on the dough network (30), reducing considerably the torque under mechanical shear stress. Yogurt-doughs expressed the negative torque values in C2, compared to control dough (control dough = 4.3 mNm ± 0.6 mNm), suggesting that the caseins and exopolysaccharides (EPS) are probably promoting flexible interactions in the dough matrix (30), reducing considerably the torque under mechanical shear stress. In opposite, curd cheese-doughs are overlapping the ones from control dough, with torque values ranging from 6.3 ± 0.6 (Cc10%) and 5.7 ± 1.2 (Cc20%) but without significant (*p* > 0.05) differences. The higher amount of curd cheese-derived protein and further interaction with flour components might be a plausible explanation for the increase in torque values, resulting in dough structuring effects.

As the heating stage proceeded (up to 95°C), the dough consistency started to increase due to the starch gelatinization, raising the torque values in C3, which is associated with the leaching out of amylose chains into the aqueous intergranular phase, consequently increasing the torque values (29). It is worth noting that as the concentration of both dairies increased in the dough matrix, a decrease in C3 values is notably observed, especially for curd cheese incorporations. Here, it might be explained by considering the functional ability of proteins to interact with amylose molecules in the dough system. Additionally, the consistency torque values in C4 were significantly reduced for curd cheese-dough, showing the stability of the starch gel, which probably can be a result of the starch dilution in the system, due to the higher levels of proteins and lipids derived from Cc incorporation. In yogurt dough, the differences observed were negligible. These findings are in line with those recently reported by Santos et al. (31), by testing the potential of chickpea and psyllium in gluten-free breadmaking.

During the cooling stage, the dough consistency represented by the C5 torque is driven by the starch retrogradation, mediated by amylose chain crystallization. This final stage depends on several variables, that can go from the nature of the starch, the presence of gelling ingredients, and the water-binding ability, to promoting a homogeneous water distribution, that can influence the bread staling and shelf-life (29, 31). Getting back to the pasting profile, at the C5 step, curd cheese doughs exhibited the lowest final consistency. This result might be attributed to the interaction of curd cheese components, such as proteins, with starch granules, which hindered the gelatinization reducing the damaged starch granules and lowering the amylose concentrations in the continuous phase (31). At the level of bread hardening during storage, it can probably constitute a quality advantage (32).

The relation between the addition of the dairy products between the impact observed on pasting properties and the possible influence on bread staling during storage were studied by multiples regression analysis. Linear correlations found are presented in the section “Dough viscoelastic behavior.”

### Steady shear flow curves

**FIGURE 2:**
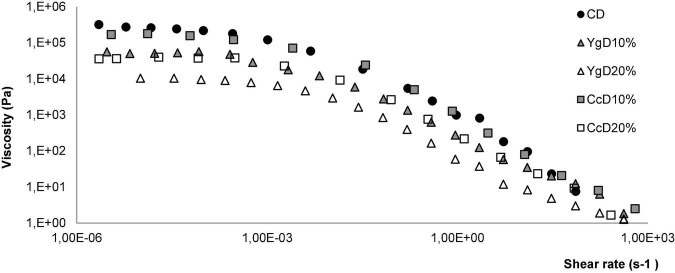
Flow behavior registered under steady shear conditions, obtained for control dough (CD) and doughs obtained with different levels of Yg (YgD10%, YgD20%) and Cc additions (CcD10%, Cc20%).

The effect of the yogurt and curd cheese additions, at different levels tested (10 and 20%, w/w), on steady shear behavior of the gluten-free doughs was evaluated, and the flow curves obtained are portraited in Figure 2. As it can be seen, all systems exhibited a typical shear-thinning behavior, that is, an initial Newtonian region with a constant viscosity at a low shear rate, followed by a viscosity decay, as the shear rate values increase. This study agrees with a recent survey focused on the utilization of different hydrocolloids (e.g., xanthan gum) and dairy proteins’ interaction with rheology properties of gluten-free bread formulations (33, 34).

The experimental data obtained were fitted well by the Carreau model (*R*^2^ > 0.977), and the main flow parameters that characterize the dough flow behavior are as follows: η_0_ – zero-shear rate-viscosity (Pa.s), γ .**_*C*_** – critical shear rate for the onset of the shear-thinning behavior (s^–1^), and s – the dimensionless parameter related to the slope of the shear-thinning region, are summarized in Table 2.

**TABLE 2:** Carreau model parameters obtained for control dough (CD) and doughs obtained with different levels of Yg (YgD) and curd cheese (CcD) additions*.

Dairies levels (%)	η_0_ (k Pa s)	γ ._*C*_ (s^–^^1^)	s (slope)	R^2^
CD	290.0 ± 8.4^a^	1.7E^–3^ ± 2.5E^–4a^	0.27 ± 0.03^a^	0.988
YgD10**%**	50.5 ± 3.4^b^	2.2E^–3^ ± 1.4E^–4a^	0.19 ± 0.01^b^	0.980
YgD20%	10.1 ± 1.8^c^	2.5E^–3^ ± 1.3E^–4a^	0.19 ± 0.01^b^	0.977
CcD10**%**	178.1 ± 6.6^d^	3.2E^–3^ ± 2.3E^–4b^	0.19 ± 0.02^b^	0.980
CcD20%	37.3 ± 4.0^e^	3.6E^–3^ ± 2.3E^–4b^	0.18 ± 0.09^b^	0.975

*Different subscript letters (a, b, c, d, e) at the same column indicate significant statistical differences at *p* ≤ 0.05 (Tukey’s test), compared with the control bread values.

As can be observed from the zero shear rate viscosity values (η_0_), higher dough viscosities were obtained for the control dough (CD), followed by CcD10% and YgD10%. According to previous works (33–35), gluten-free doughs obtained with hydrocolloids, such as xanthan gum addition, showed high viscosities due to the complex aggregates formed by strong and stiff molecular linkages, reducing the flow behavior. It has been reported that higher viscosity might retain bubbles in the batter, but it also restricts the dough expansion during fermentation and consequently, the breadmaking performance (36, 37).

As the concentration of both dairies increase in the dough matrix, a marked decrease in dough viscosity (η_0_) was registered, varying from 37.3 k Pa s (CcD20%) to 10.1 k Pa s (YgD20%) representing a reduction of around 87.2 and 96.5%, compared to control dough (CD). The behavior observed might suggest that the addition of both dairies promoted a considerable dilution effect of the density of the dough matrix macromolecule interaction, reducing the dough viscosity. The observed decrease in viscosity can probably be explained by the presence of yogurt-derived exopolysaccharides (EPS, produced by lactic acid bacteria), and their water-binding and holding abilities (38), reducing the stiffest linkage molecules of the dough matrix. These findings agree with other author’s surveys, by studying the interaction of hydrocolloids with caseins and albumins on GF bread (39) and whey protein on GF cakes (40) technological performances, reporting a marked reduction in dough rheology parameters.

As for critical shear rate, significant differences can be noticed, comparing yogurt and curd cheese dough systems with control: for the former, viscosity began to decrease at around 2.2 × 10^–3^ s^–1^, and for the latter, the breakdown occurred at around 3.2 × 10^–3^ s^–1^, comparing to 1.7 × 10^–3^ s^–1^ of control dough. This behavior suggests that, although the addition of both dairies has promoted the dilution effect on a stiffer complex aggregate, reducing the molecular links extends the first Newtonian plateau. Regarding the dough flow indexes (slope), both dairy’s addition promoted a slight but significant decrease (*p* < 0.05), compared to control dough, from 0.27 to 0.19. This means that the flow index of the shear thinning region increased from 0.73 to 0.81, in line with the expansion of the first Newtonian plateau, being this flow closer to Newtonian, therefore less shear thinning, from a less structured material.

### Dough viscoelastic behavior

The supplementation of yogurt and curd cheese (10 and 20%, w/w) on gluten-free doughs was also assessed by its influence on viscoelastic behavior, in terms of elastic (G′) and viscous (G″) moduli changes, by oscillatory frequency sweep measurements.

**FIGURE 3:**
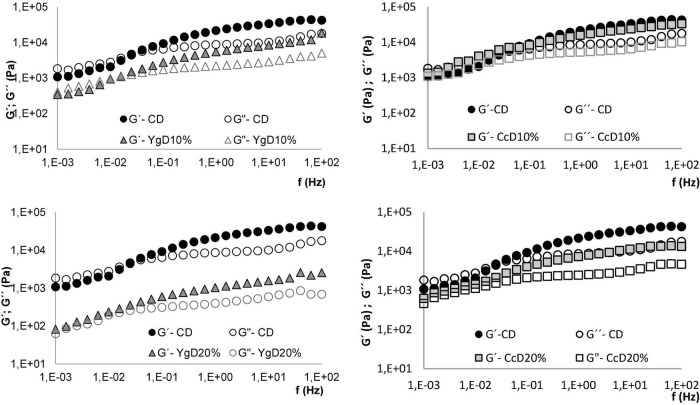
Changes in gluten-free dough viscoelastic behavior, elastic (G′) and viscous (G″) moduli, promoted by different levels of yogurt and curd cheese additions (YgD and CcD at 10 and 20%) compared to control dough (CD).

The mechanical spectra of control dough (CD) and doughs obtained with yogurt (YgD) and curd cheese additions (CcD) are depicted in Figure 3.

From Figure 3, it can be noticed that even though both dairies lowered the viscoelastic functions of the dough rheology behavior, the effect promoted by yogurt addition was greater compared to curd cheese incorporation, expressing lower elastic (G′) and viscous (G″) moduli values than control dough (CD). This behavior suggests a formation of weaker structures-like batter with greater plasticity, with a remarkable effect at 20% of both product’s addition (34). These findings might indicate the formation of a more flexible macromolecule’s linkages, because of the water-binding and holding abilities of the dairies added (38), reducing the stiffness of the dough network. These results are in line with those previously observed at flow curve measurements.

Evaluating in more detail the viscoelastic profiles obtained at low-frequency values, the systems display different behaviors. Starting from control dough (CD) and yogurt-dough systems, the values of G″ are higher than G′, exhibiting a typical viscoelastic fluid behavior (40), characteristic of an entangled network structure (41, 42). Nevertheless, the dominance of the G′ over the G″ occurred with the frequency increase, expressing a typical weak-gel behavior (42), with high-frequency dependence (38). It can also be noticed that the frequency values of the G′ crossing over the G″ were reduced by the yogurt addition to dough, varying from 0.025 Hz for CD to 0.004 Hz for YgD20% (higher level tested). The reinforcement of the molecular linkages on the dough matrix might be considered to explain this behavior, which agrees with the pasting properties and flow index results discussed above.

When it comes to the influence of curd cheese, although a typical weak-gel structure can be observed (41), the G′ values were greater than the G″ over the whole frequency range, indicating the prevalence of the viscoelastic behavior (42), with high-frequency dependence (38).

These results might indicate a formation of a new protein network aided by denatured whey protein interaction, derived from Cc addition, building up a more complex and flexible dough network. It is well known that the viscoelasticity of the gluten-free dough depends strongly on intra- and intermolecular interactions, which means a network with a desirable viscoelastic behavior is required for a good breadmaking performance. The chemical composition and functional properties of both yogurt and curd cheese-derived macromolecules and their functional interactions are probably accounting for the changes in dough viscoelastic behavior, acting on the system through the following possible three mechanisms:

i) Enhancing the orientation/disentanglement of the macromolecule’s interaction in the dough matrix, under oscillatory conditions (43), by improving the flexibility properties;
ii) Reducing the stiffness of the dough network formed by xanthan gum, generating a more flexible protein chain (33);
iii) Strengthening of the dough molecules’ linkages, by extra protein and exopolysaccharides interaction, gives more stability to the dough network (33, 44).

These findings agree with those reported by other authors (33, 45) assessing the influence of several hydrocolloids (e.g., xanthan gum) and different protein sources (e.g., whey protein) on rheology properties of gluten-free bread formulas.

In short, these results suggest that the dairy products are potential baking ingredients to enhance the dough foaming capacity and further ability of gas bubbles retaining, which are required to achieve the desired technological properties in terms of bread texture and volume (45).

### Gluten-free bread quality attributes

The quality of the derived gluten-free bread samples, based on post-baking parameters, such as bread crumb texture (N) and staling rate (N/h), moisture (%), bake loss (%), and specific bread volume, was evaluated.

The bread crumb textures of the control and dairy gluten-free bread samples were assessed by texture profile analysis applying a puncture assay. Bread staling kinetic was linearly described (*R*^2^ > 0.974) as a function of time, following the firmness progress during the 4 days of storage time (96 h). The linear parameters presented in Table 3 reflect the impact of both yogurt and curd cheese additions on bread staling rate (A, the slope) and initial firmness (B, the interception).

**TABLE 3:** Bread stalling parameters: A- bread staling rate (N/h) and B- bread firmness (N), obtained for control bread (CB) and breads with different levels of Yg and Cc tested (YgB, CcB)*.

Yg/Cc levels	B - Initial firmness (N)	A- Staling rate (N/h)	R^2^
CB	2.50 ± 0.13^a^	0.044^a^	0.997
YgB10%	0.95 ± 0.10^b^	0.026^b^	0.975
YgB20%	0.82 ± 0.20^b^	0.015^b^	0.993
CcB10%	1.90 ± 0.13^c^	0.022^c^	0.981
CcB20%	1.12 ± 0.10^d^	0.012^cd^	0.972

*Different subscript letters (a, b, c) within the same column indicate significant statistical differences at *p* ≤ 0.05, (Tukey’s test), compared with the control bread parameters.

It can be observed that both yogurt and curd cheese promoted a significant (*p* ≤ 0.05) positive impact to improve the bread crumb softness: control bread firmness was higher than both dairy levels tested, varying from 2.50 N for control bread (CB) to 0.82 N for yogurt (YgB20%) and 1.12 N for curd cheese (CcB20%), at higher levels of incorporation, representing a decrease of 67 and 55% of crumb firmness, respectively.

It is worth noting that the higher bread firmness values of control bread are consistent with the higher viscosity and viscoelastic moduli values exhibited for control dough. These effects are probably associated with the stiffer dough linkages between xanthan gum and non-gluten proteins and other flour components, which consequently resulted in less dough expansion and harder breads.

Similar findings were reported by Demirkesen et al. (33), where the increase of bread crumb firmness by xanthan gum addition in gluten-free breadmaking was noticed.

Furthermore, the bread staling rate values were substantially lower than those obtained for CB, ranging from 0.015 N/h for yogurt (YgB20%) and 0.012 N/h for curd cheese (CcB20%) bread samples, compared to 0.044 N/h of CB, a considerable (*p* < 0.05) reduction about 67 and 70% of staling, respectively. Comparable results were previously reported, emphasizing the impact of the dairy powders (10) and fresh dairy product additions (14) as technological potential ingredients on bread quality post-baking attributes and nutritional and sensory bread properties.

Bread staling during the storage time is a consequence of moisture loss, followed by the starch crystallization/retrogradation phenomena, increasing the crumb firmness values (45).

The increase of moisture content either for yogurt or curd cheese bread was proportional to the level of dairies added, varying from 42.0 for CB to 50.0% and 51.0% for YgB20% and CcB20%, corresponding to an increase of around 20% in both bread types.

Water is the most important plasticizer in food structure, acting as a chain polymer lubricant, reducing stiffer linkage forces by increasing the flexibility of the dough structure (46).

Additionally, the yield of both dairy bread types was markedly improved by around 30% compared to CB, which might be associated with the lesser weight loss during baking, due to the improved water holding capacity by dairy polysaccharides and protein sources addition (47).

Improved yogurt-bread properties can probably be linked to the derived exopolysaccharides, since their ability of binding water and retain moisture has been associated with slowing down the starch crystallization and bread staling rate (30).

As for curd cheese bread, the functionality of the whey protein can be considered to explain these good results of bread texture and staling rate, once their water binding ability and moisture retaining might slow down the starch retrogradation and bread hardening (18, 48). On the other hand, these results suggest that both casein and the denatured whey protein might have the ability to relax the macromolecules interaction, improving the dough foaming capacity and stability to retain the air bubbles, therefore, improving the bread properties (42, 48).

Based on specific bread volume, a significant improvement was registered for both dairy bread types obtained, varying from 1.80 cm^3^/g for CB to 2.50 cm^3^/g for YgB20%, and 2.60 cm^3^/g for CcB20%, with an increase of around 40%. These improvements in specific bread volume are proportional to the increase of dairies in the dough matrix and the effects can be linked to the enhanced water-holding capacity, which makes the starch molecules more prone to forming a uniform and more flexible starch-protein matrix, which further enhanced during the baking process (30, 31, 49).

**FIGURE 4:**
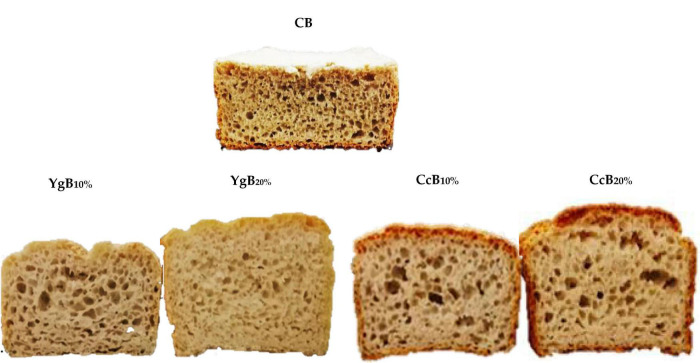
Comparison of yogurt and curd-cheese-derived gluten-free bread at different levels of incorporation (YgB and CcB10% and 20%) with control bread (CB, without dairies addition).

Similar findings were reported by Sahagún and Gómez (47) evaluating the effect of other protein sources on the physicochemical and quality properties of GFB. The contribution of the yogurt-derived casein and exopolysaccharides, as well as the curd cheese-derived whey proteins, cannot be excluded as they are known for being capable of building a structured starch-protein matrix (48, 50, 51) and can build up a bread structure with uniform gas cell distribution, which results in better bread volumes (Figure 4).

Figure 4 portrays the obtained bread samples with different levels of yogurt (YgB10% and YgB20%) and curd cheese (CcB10% and CcB20%) addition, in comparison to control bread (CB).

The above-described results allow stating that the yogurt and curd cheese as bakery ingredients promoted a markedly increase in bread crumb softness as well as delayed staling rate of the derived-gluten-free bread, leading to an extension of the shelf-life, which is an important industrial advantage.

Linear correlations were studied between dough rheology properties and bread quality attributes to find out the main variables involved in the breadmaking process.

### Relationship between bread quality parameters and dough rheology properties

**FIGURE 5:**
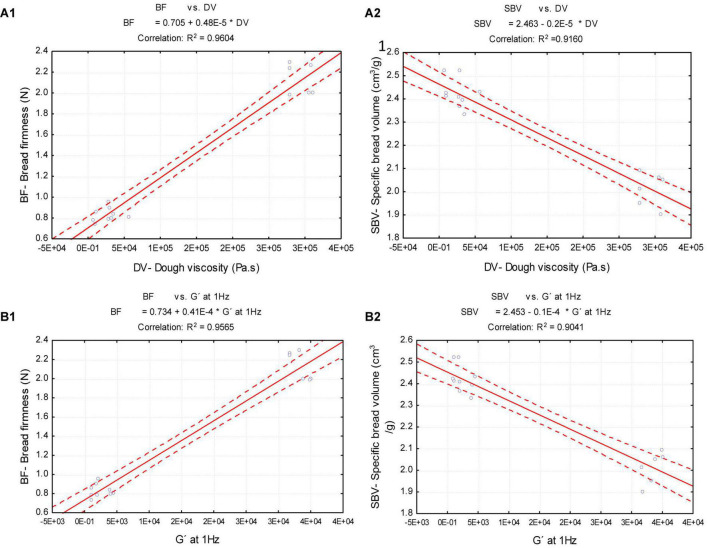
Linear correlation between bread firmness (BF) and specific bread volume (SBV) with dough viscosity (DV) and elastic modulus at 1 Hz (G′): **(A1)** BF vs. DV; **(A2)** SBV vs. DV; **(B1)** BF vs. G′ **(B2)** SBV vs. G′.

The results presented in this work suggested a strong relationship between bread quality parameters and the promoted changes in dough rheology properties by dairy products supplementation. Therefore, linear correlations (*R*^2^ > 0.9041) between the bread firmness (BF) and specific bread volume (SBV) with steady shear (DV-dough viscosity) and oscillatory (G′- elastic modulus at 1 Hz) values of dough rheology, were studied. The linear correlations obtained are illustrated in Figure 5.

As it can be observed from Figures 5A1–B2, while the control dough (only with xanthan gum) exhibited higher dough viscosity (Figures 5A1,A2: DV) and elasticity (Figures 5B1,B2: G′ at 1 Hz) values, the bread firmness (Figures 5A1,B1: BF) and the specific bread volume (Figures 5A2,B2: SBV) were not that high. Previous works (33, 52) reported that although xanthan gum had the most pronounced effect on viscoelastic properties of the dough, the bread texture and volume were negatively affected.

In opposite, lower values of dough viscosity (Figures 5A1,A2: DV) and elastic modulus (Figures 5B1,B2: G′ at 1 Hz), by adding both dairies to the dough, resulted in softer breads (Figures 5A1,B1: BF), with higher specific bread volumes (Figures 5A2,B2: SBV) than control bread.

These findings disagree with those obtained by other researchers (33, 52) evaluating different gums, and emulsifiers on gluten-free bread formulations, where higher dough viscosity and elastic values resulted in lower firmness values of the bread. It suggests that the quality of the gluten-free bread depends strongly on the type of the ingredients used and the interactions between the macromolecules into play, to mimic the structure-building like gluten matrix.

The staling rate is a consequence of the moisture migration from the crumb to the crust, water distribution, and the starch crystallization/retrogradation phenomena, which increase the crumb firmness during the storage time (45). The lower bread staling rate obtained might be associated with the impact of the dairy products on pasting properties, particularly on starch gelatinization (SG) performance, cooking stability (CS), and further on final consistency (FC). A strong linear correlation between starch gelatinization (A: *R*^2^ = −0.930), cooking stability (B: *R*^2^ = −0.910), final consistency (C: *R*^2^ = −0.966), and the dairy product levels tested, supports the hypothesis that the possible interaction of the starch with the components derived from dairy products might be a valid explanation to the decreased starch performance and the lower stability of the starchy gel formed. Furthermore, it ended up in a considerable reduction of the final dough consistency, which is possibly related to the lower amylose molecules to form the recrystallization chains (53, 54).

The reduction of the dough’s final consistency was linked to the lowering of bread staling during storage by Santos et al. (32). Our findings are in line with those reported by Santos et al. (32), where the good linear correlation (D: *R*^2^ = - 0.944) found between the staling rate and the addition of the dairy products might suggest a high potential of these products to reduce bread hardening during storage.

**FIGURE 6:**
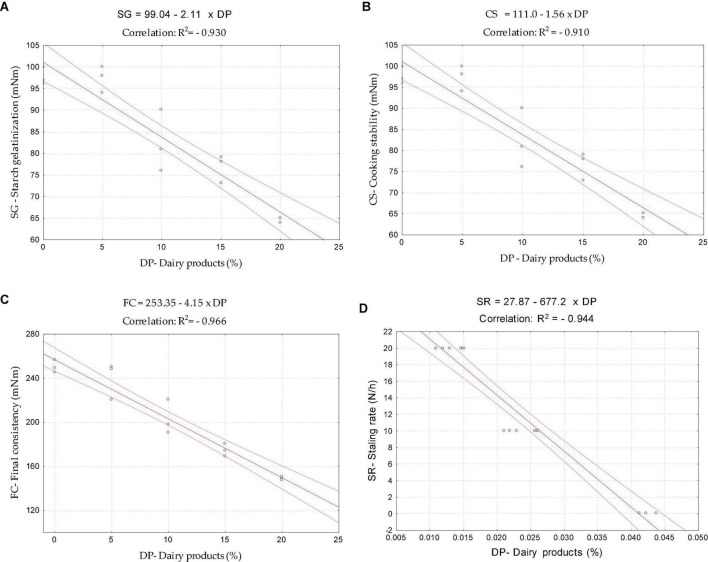
Linear correlation between starch gelatinization (SG), cooking stability (CS), final consistency (FC), and staling rate (SR) with the addition of both dairy product (DP): **(A)** SG vs. DP; **(B)** CS vs. DP; **(C)** FC vs. DP; **(D)** SR vs. DP.

These linear correlations (Figures 6A–D) support the results presented along with this work, showing a strong correlation between bread quality parameters obtained and dough rheology changes promoted by the addition of both dairy products.

## Conclusion

Gluten-free bread formulations, with different levels of yogurt and curd cheese additions, were evaluated using dough rheology measurements and baking quality parameters.

Based on the obtained results, it was found that the derived gluten-free dough systems presented rheology properties typically of weak gels, with values of storage G’ and loss G” moduli significantly frequency-dependent. Additionally, the dough systems exhibited a non-Newtonian and shear-thinning flow behavior, that was further accentuated by the presence of dairy products, decreasing the apparent viscosity and the flow index. The possible dilution of the starch by the addition of both dairies might also cause a decrease in dough consistency during pasting mixing. The dough systems showed a batter-like behavior, which is the desired property in gluten-free breadmaking.

Although, the dough rheology properties were markedly changed as the concentration of both dairies’ incorporations increased into the dough matrix, such effects promoted a significant improvement in the overall quality of the dairy-derived bread.

Strong linear correlations between bread firmness, specific volume with apparent viscosity and viscoelastic functions, and the pasting properties with dairy products and staling rate were found, supporting the good findings obtained. The positive effects observed were proportional to the increased levels of both dairies in the gluten-free dough.

In short, the dairy products tested showed to be promising alternative bakery ingredients to overcome the technological challenge of gluten absence, resulting in dairy bread types with desirable attributes in terms of softer texture, slowly staling, high volume, and appealing appearance, compared to the control bread.

Considering the technological knowledge generated by this research and the good quality of the developed baking goods, the establishment of collaborations with industrial partners would be important to be performed, in order to take the developed products to a commercial level.

## Data availability statement

The raw data supporting the conclusions of this article will be made available by the authors, without undue reservation.

## Author contributions

CG: conceptualization, methodology, investigation, data curation, and writing – original draft preparation. AR and IS: project administration, investigation, supervision, validation, and writing – review and editing. All authors have read and agreed to the published version of the manuscript.
